# Completeness and timeliness of notifiable disease reporting: a comparison of laboratory and provider reports submitted to a large county health department

**DOI:** 10.1186/s12911-017-0491-8

**Published:** 2017-06-23

**Authors:** Brian E. Dixon, Zuoyi Zhang, Patrick T. S. Lai, Uzay Kirbiyik, Jennifer Williams, Rebecca Hills, Debra Revere, P. Joseph Gibson, Shaun J. Grannis

**Affiliations:** 1 Indiana University, Richard M. Fairbanks School of Public Health, 1050 Wishard Blvd, RG 5000, Indianapolis, IN 46202 USA; 2 Regenstrief Institute, Center for Biomedical Informatics, 1101 W 10th St, Indianapolis, IN USA; 3 Department of Veterans Affairs, Health Services Research & Development Service, Center for Health Information and Communication, 1481 W. 10th St, 11H, Indianapolis, IN USA; 4 Department of BioHealth Informatics, School of Informatics and Computing, Indiana University, 535 W Michigan St, Indianapolis, IN 46202 USA; 5 University of Washington, School of Public Health, 1107 NE 45th St, Suite 400, Box 354809, Seattle, WA 98195-4809 USA; 6 Marion County Public Health Department, 3838 N Rural St, Indianapolis, IN 46205 USA; 7 Indiana University, School of Medicine, 3410 10th St, #6200, Indianapolis, IN USA

**Keywords:** Health information exchange, Disease notification, Public health surveillance, Completeness, Timeliness, Electronic laboratory reporting

## Abstract

**Background:**

Most public health agencies expect reporting of diseases to be initiated by hospital, laboratory or clinic staff even though so-called passive approaches are known to be burdensome for reporters and produce incomplete as well as delayed reports, which can hinder assessment of disease and delay recognition of outbreaks. In this study, we analyze patterns of reporting as well as data completeness and timeliness for traditional, passive reporting of notifiable disease by two distinct sources of information: hospital and clinic staff versus clinical laboratory staff. Reports were submitted via fax machine as well as electronic health information exchange interfaces.

**Methods:**

Data were extracted from all submitted notifiable disease reports for seven representative diseases. Reporting rates are the proportion of known cases having a corresponding case report from a provider, a faxed laboratory report or an electronic laboratory report. Reporting rates were stratified by disease and compared using McNemar’s test. For key data fields on the reports, completeness was calculated as the proportion of non-blank fields. Timeliness was measured as the difference between date of laboratory confirmed diagnosis and the date the report was received by the health department. Differences in completeness and timeliness by data source were evaluated using a generalized linear model with Pearson’s goodness of fit statistic.

**Results:**

We assessed 13,269 reports representing 9034 unique cases. Reporting rates varied by disease with overall rates of 19.1% for providers and 84.4% for laboratories (*p* < 0.001). All but three of 15 data fields in provider reports were more often complete than those fields within laboratory reports (*p* <0.001). Laboratory reports, whether faxed or electronically sent, were received, on average, 2.2 days after diagnosis versus a week for provider reports (*p* <0.001).

**Conclusions:**

Despite growth in the use of electronic methods to enhance notifiable disease reporting, there still exists much room for improvement.

## Background

Surveillance is the cornerstone of public health practice [1, 2]. Traditionally, health departments wait for hospital, laboratory or clinic staff to initiate most case reports [3]. However, such passive approaches are known to be burdensome for reporters, producing incomplete and delayed reports, which can hinder assessment of disease in the community and potentially delay recognition of patterns and outbreaks [4–6].

Modern surveillance practice is shifting toward greater reliance on electronic transmission of disease information. The adoption of electronic health record (EHR) systems and health information exchange (HIE) among clinical organizations and systems [7–9], driven by policies like the ‘meaningful use’ program in the United States [10], is creating an information infrastructure that public health organizations can leverage for improving surveillance practice [11–14].

According to the U.S. Centers for Disease Control and Prevention (CDC), health departments currently receive up to 67% of their total laboratory-based reports for notifiable diseases as electronic laboratory reports (ELR) [13]. However, provider-based case reporting continues to be largely paper-based via fax machines [15, 16].

Policymakers who published the most recent iteration of the meaningful use requirements [17] envision that EHR systems and HIE networks supply infrastructure that supports electronic submission of treatment, corollary results and other details from providers that are not available from laboratory information systems. In this model, providers could receive automatically generated electronic case reporting forms through their EHR, which could be completed and sent to local health departments for case investigation.

The CDC is exploring a new technology platform to support this kind of functionality for commercial EHR systems [18]. However, it is unknown whether such a platform would help to improve reporting rates, timeliness and completeness.

In this article, we describe patterns of reporting as well as data completeness and timeliness for traditional, passive reporting of notifiable disease. This work provides a baseline to assess an intervention we are testing that leverages the emerging EHR and HIE infrastructure to provide accurate, timely surveillance [19, 20]. The goal of the ongoing trial is to make it “easier to do the right thing” [21] (i.e., report to public health) by pre-populating reporting forms using an expanded set of data extracted from EHRs, thus reducing manual data entry to the extent possible for clinical staff. This paper describes dimensions of case reporting quality (e.g., completeness and timeliness) prior to the intervention.

## Methods

Our study was performed in the context of the Indiana Health Information Exchange, a large HIE network that delivers laboratory results, radiology results and other clinical messages to providers since 1999 [22, 23]. Using components within the HIE information infrastructure, including the Notifiable Condition Detector [24, 25], this study provides baseline data for a future intervention which will pre-populate the official Indiana State Department of Health communicable disease reporting form with patient demographics, notifiable disease confirmatory test results, and provider information. The pre-populated form will be delivered electronically to the provider using the HIE network. A detailed description of that study protocol is available [26].

The study examined case reporting for seven representative diseases of varied incidence and consequence that are commonly investigated by local health department staff in Indiana and include: Salmonellosis, Hepatitis C, Hepatitis B, Chlamydia, Gonorrhea, Syphilis and Histoplasmosis. As in most states, Indiana clinicians and laboratories are required to report each reportable disease case they encounter.

Prior to deploying the intervention, we gathered information from all electronic and paper-based notifiable disease reports submitted by both providers and laboratories to the Marion County Public Health Department in Indianapolis, Indiana, which had a mid-2014 population of 934,243. Faxed laboratory reports for reportable diseases (Faxed LR) were sent by laboratory technologists as well as infection preventionists, and ELRs were delivered through the Indiana Health Information Exchange (HIE-ELR). Provider reports are faxed to the local health department by individuals working in clinics and hospitals, including infection preventionists who are tasked with reporting notifiable disease information for a health system. To assure reasonable power for a future evaluation, we varied the time period for collecting data for each disease, based on its prevalence. We gathered reports for highly prevalent diseases (e.g., Chlamydia, Gonorrhea) over a 3-month period (May–July 2012), moderately prevalent diseases for 6–8 months (Syphilis, December 2011–July 2012; Hepatitis C, February 2012–July 2012) and less prevalent diseases (e.g., Histoplasmosis, Salmonellosis, Hepatitis B) were gathered over 2 years (August 2010–July 2012).

Reports from the various sources were grouped into unique disease episodes (cases) for the same patient using CDC case definitions [27]. Given the lack of a master person index (MPI) at the health department [28], we linked available patient identifiers (e.g., first name, last name, gender, date of birth, phone number) using probabilistic record-linkage, with minimal human review to resolve questionable matches, to create unique patient identifiers [29]. A case was therefore composed of a set of reports from one or more sources for the same patient for the same disease episode.

Key information necessary for case investigation by health department staff was extracted from each paper or electronic report and collated by disease (see Table 2). The data were extracted manually by trainees in public health informatics (including UK and PTL) and cross-validated for accuracy whereby one trainee would verify the data entered by another trainee. Data fields were recorded as blank when the information was absent in a given report. Dates were entered as they were either timestamped (e.g., automatically by information systems) or hand written onto reports, even when the received date at the public health agency occurred before the date of the laboratory test or diagnosis as this reflects actual data in the real-world case files. While captured in the study database, negative time differences and values outside of expected ranges (e.g., outliers) were excluded during analysis.

Reporting rates were calculated by dividing the number of unique cases with at least one report from a given data source (e.g., Provider, Faxed-LR or HIE-ELR) by the total number of unique cases observed during the same time period. Observed cases include those reported from any of the three sources, as well as cases managed by public health clinics, which do not always produce a provider, faxed-LR or HIE-ELR case report but instead enter case information directly into the health department’s case management system. Provider reports, for the purpose of analysis, consisted of both the official Communicable Disease Report forms created by the state health agency for use by providers as well as other documentation (e.g., scanned medical charts) that is faxed to the health department from clinics. In addition, although we separated HIE-ELR and faxed-LR for some analyses, we also combined reports from laboratory sources into an aggregate “all laboratory” source since health departments generally don’t distinguish among these in routine operations. Finally, some cases contained multiple reports of the same type. For example, both the infection control staff at a hospital and the patient’s primary care physician might submit a provider report. In other cases, the laboratory directly submitted a faxed-LR to the health department and an HIE-ELR was delivered via the Indiana HIE.

To compare reporting rates, we utilized McNemar’s test. Cases with provider reports were grouped into dyads based on the other report types included for that case (e.g., Provider vs. Faxed-LR, Provider vs. HIE-ELR) and McNemar’s test was used to assess whether the two reporting rates were significantly different from one another.

Completeness was measured as the percentage of data fields containing values at the individual report level. The completeness of case reports from four sources was compared: provider, faxed-LR, HIE-ELR and all laboratory sources (i.e., faxed-LR or HIE-ELR). The analyzed fields were selected by public health practitioners as being critical for case investigation and reporting to state health authorities as well as the CDC. Therefore each case report is expected to contain this minimum set of data.

Analysis of timeliness focused on the difference, in calendar days, between the test result date and the date the case was either reported by the data source (e.g., date stamped by a fax machine or computer system) or when the report was received by the local health department (e.g., ink stamp applied by staff) if the report date was missing. Reports were grouped and compared by source. Negative values, where the received date occurred before the test date, accounted for a fraction of a percent and were therefore removed prior to analysis.

The methods for establishing completeness of report data elements and the timeliness of reports were adapted from our prior work [30, 31]. Comparisons of completeness and timeliness across the four data sources were performed using generalized linear models (GLM). For completeness, a binomial GLM was conducted separately for each field, where the clustering effect of completeness from the four data sources for the same patient was accounted for using generalized estimating equations. For timeliness, the difference, measured as a count of days, between the date of the laboratory test and the date the result was reported to public health was modeled using a negative binomial GLM, where the clustering effect of multiple cases for the same patient were accounted for using generalized estimating equations. All analyses were performed using SAS version 9.4 (Carey, NC).

## Results

A total of 13,269 reports representing 9034 unique cases for 8353 unique patients were gathered from health department records. The dataset represents all reports to the health department during the respective baseline time periods for the seven diseases.

### Reporting rates

**Fig. 1:**
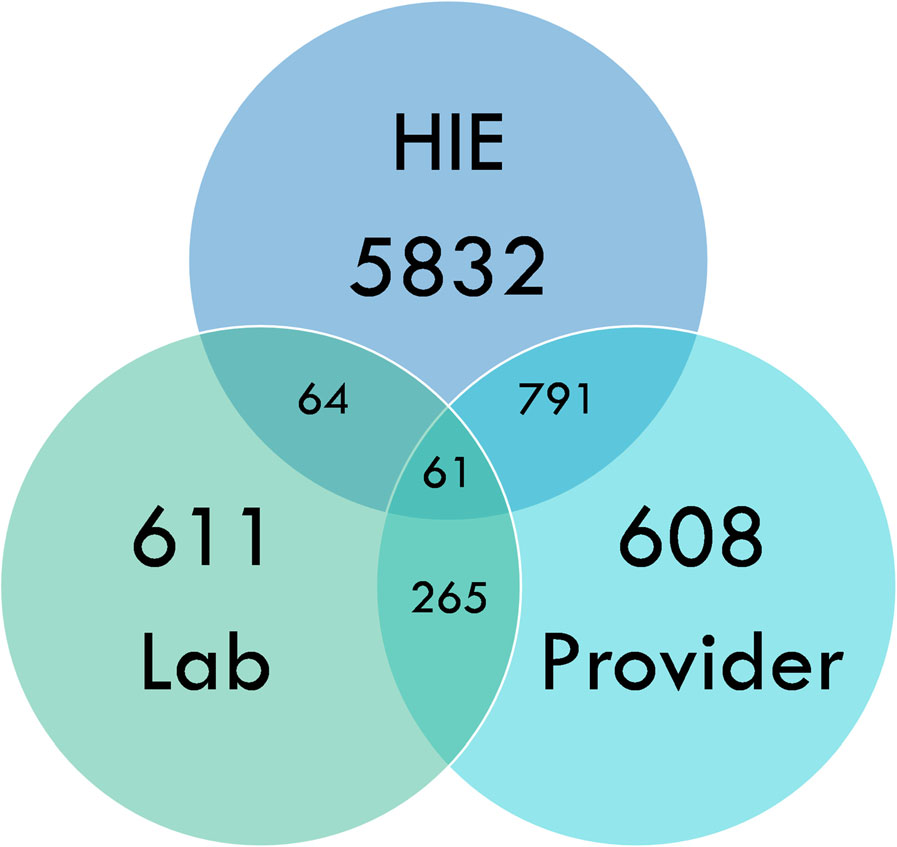
Venn diagram depicting the count of unique cases with at least one report from one of the following data sources: Provider, Laboratory or Health Information Exchange (HIE). The overlapping sections of the diagram indicate how many unique cases contained at least one report from two or more data sources

Providers submitted 2130 reports representing 1725 cases for 1615 patients; 1324 faxed-LR represented 1001 cases for 945 patients; and 7640 HIE-ELR reports represented 6748 cases for 6266 patients. These figures translate into the following reporting rates: 19.1% for providers; 11.1% for faxed-LR; and 74.7% for the HIE-ELR. Examining cases with at least one faxed-LR or HIE-ELR (*N* = 7624) results in an overall “all laboratory” reporting rate of 84.4%. Figure 1 is a Venn diagram depicting the count of unique cases with at least one report from one data source. The diagram also visualizes the amount of overlap among data sources, or where more than one data source contributes a report to a unique disease case. Most reports came from the HIE-ELR with similar proportions contributed by providers and laboratories via fax (e.g., Faxed-LR). Thirteen percent of cases (*N* = 1181) included multiple reports from the same source (e.g., two provider reports).

Reporting rates varied by disease (Table 1). Providers reported nearly half of known cases for common sexually transmitted infections like chlamydia and gonorrhea (although not syphilis which was 6.3%, *p* < 0.01) while reporting only one-third of known cases of conditions like salmonellosis. Provider reporting rates per disease ranged from 0.5% [acute hepatitis B] to 44.4% [chlamydia] (*p* < 0.01). When faxed-LR and HIE-ELR were combined, representing the union of cases that contained at least one laboratory report, reporting rates were significantly higher, ranging from 63.1% [gonorrhea] to 99.8% [acute hepatitis B] (*p* < 0.01).

**Table 1:** Reporting rates^a^ by disease and source, 2010–2012

Disease	Number of cases	Provider reporting rate [reference]	Faxed-LR reporting rate	HIE-ELR reporting rate	Faxed-LR or HIE-ELR reporting rate
Chlamydia	2605	44.4%	23.1%*	48.4%*	69.9%*
Histoplasmosis	73	42.5%	15.1%*	57.5%	69.9%*
Gonorrhea	810	36.5%	20.0%*	44.8%*	63.1%*
Salmonellosis	246	30.1%	23.2%	68.7%*	84.1%*
Hepatitis C	1137	10.6%	10.7%	80.6%*	88.4%*
Syphilis	445	6.3%	8.3%	65.4%*	71.7%*
Hepatitis B	3718	0.5%	0.2%*	99.7%*	99.8%*

*HIE-ELR* Electronic laboratory report from health information exchange, *Faxed-LR* Faxed report directly from a laboratory

*p < 0.01 for pairwise comparisons where the reference was the provider reporting rate

^a^The reporting rates (%) for each data source are displayed as the proportion of cases for the disease group which contained at least one report from that source. The total n includes de-duplicated cases reported by providers, laboratories, or the HIE or known to the public health agency because the case presented directly in a public health agency clinic

**Fig. 2:**
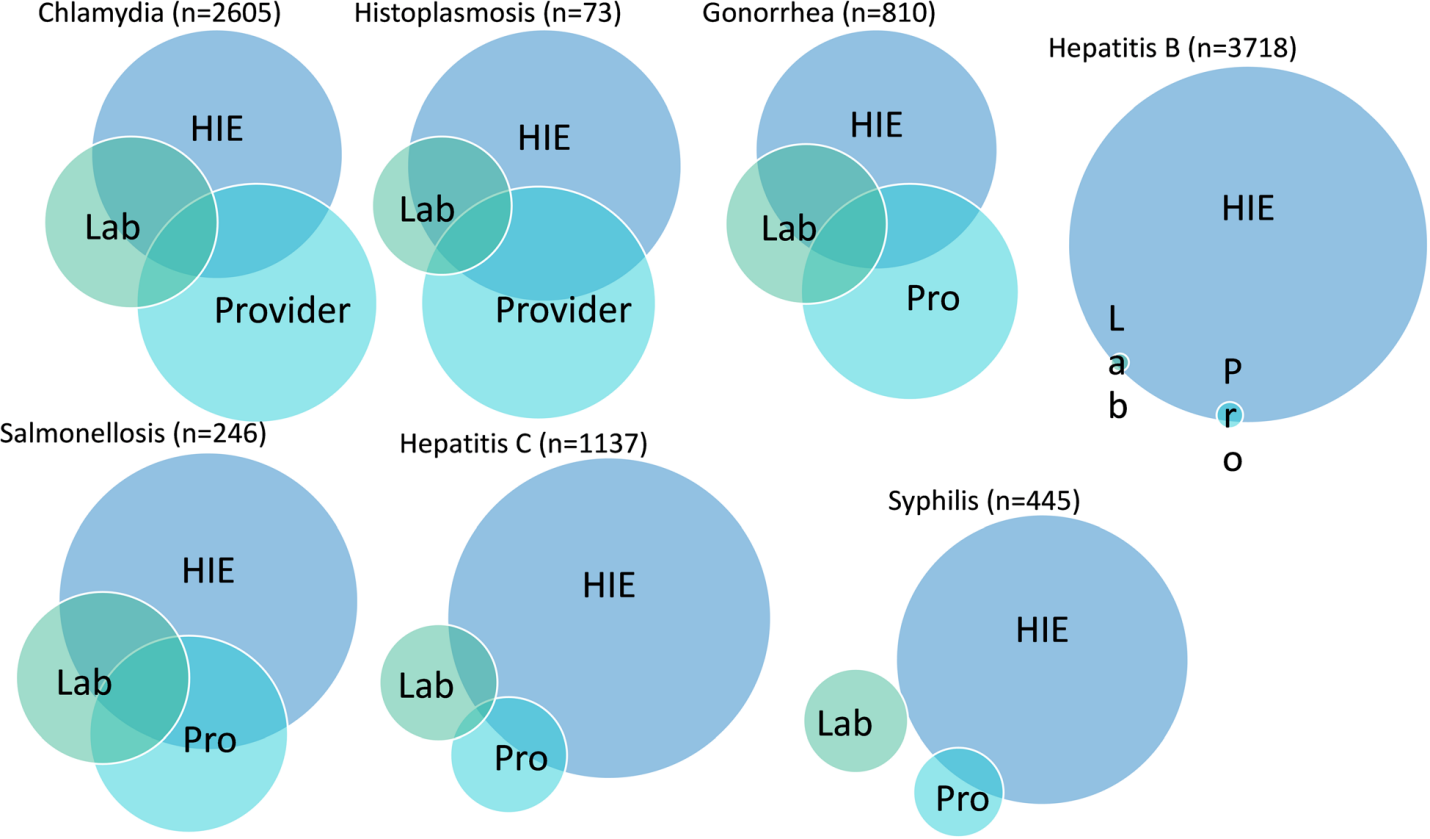
A series of Venn diagrams, stratified by disease, depicting the relative proportion of unique cases with at least one report from one of the following data sources: Provider, Laboratory or Health Information Exchange (HIE). The overlapping sections of the diagram indicate the proportion of unique cases for a given disease contained at least one report from two or more data sources

The Venn diagram in Fig. 2 visualizes reporting rates by disease in combination with data sources. Overlap among data sources is lowest for three conditions: acute Hepatitis B, Hepatitis C and syphilis. Higher overlap exists for gonorrhea, chlamydia and histoplasmosis.

### Completeness

Table 2 summarizes the completeness of data fields for provider, faxed-LR, HIE-ELR and “all laboratory” reports. With respect to provider reports, completeness ranged from 45.3% [provider zip code] to 100% [patient last name]. With respect to reports from laboratory sources, completeness used the entire range of values from 0.5% [ethnicity] to 100% [patient last name]. Some fields, such as patient name and patient date of birth, were similar in completeness across all sources. Several fields, including patient address, patient phone, ethnicity and provider address, were more complete in provider reports when compared to the laboratory report groups (*p* <0.001). Laboratory-based reports were more complete for just three fields: patient sex, provider name and identification of the laboratory test performed (*p* <0.001).

**Table 2:** Percent complete by field and data source

Data Element	Proportion of Cases with Provider Reports^a^ *N* = 1725 [reference]	Proportion of Cases with Faxed Laboratory Reports *N* = 1001	Proportion of Cases with HIE-ELR *N* = 6748	Proportion of Cases with Any Laboratory Report *N* = 7624
Patient’s Last Name	100.0	100.0	100.0	100.0
Patient’s First Name	99.9	100.0	100.0	100.0
Patient’s Date of Birth	96.5	100.0	96.8	97.2
Patient’s Sex	90.2	96.9*	100.0*	99.7*
Patient’s Street Address	87.0	57.2*	76.4*	74.9*
Patient’s Zip Code	83.8	57.4*	74.9*	73.6*
Patient’s Phone Number	78.3	65.7*	75.7	75.3
Patient’s Race	77.9	0.0*	73.6	65.2*
Physician’s Last Name	72.2	90.6*	99.2*	98.2*
Physician’s First Name	70.1	84.9*	82.7*	83.1*
Lab Test Performed	68.8	99.4*	100.0^*^	99.9*
Physician’s Phone	66.5	90.4*	37.5*	44.9*
Physician’s Address	64.7	95.6*	41.2*	48.8*
Patient’s Ethnicity	56.4	0.0*	0.0*	0.0*
Physician’s Zip Code	45.3	95.5*	25.5*	34.9*
Average	77.2	75.7	72.2	73.1

*HIE* Health information exchange, *ELR* Electronic laboratory report

*p < 0.001 for pairwise comparisons where provider report field completeness was the reference

^a^Provider report field completeness was used as the reference for calculating the χ^2^ goodness of fit test statistic

### Timeliness

The timeliness with which reports were submitted to the health department varied by source as summarized in Table 3. The most timely data source was the HIE with an average of 2.0 days (median 1 day) between when the test was performed and receipt of the case report by the health department. Laboratory reports faxed to the health department, or sent electronically via manual upload to an online reporting system operated by the state health department, were the next most timely with an average of 3.6 days (median 2 days). Provider reports were submitted an average of 10.5 days after diagnosis with a median of 5 days.

**Table 3:** Timeliness by data source

Data Source	Total N	Mean # days	Median # days	Max # days	*P*-value for χ^2^
Provider	1878	10.5	5	375	Reference
Faxed-LR	1142	3.6	2	367	<0.0001
HIE-ELR	7393	2.0	1	320	<0.0001
Any Laboratory^a^	8535	2.2	1	367	<0.0001

*HIE* Health information exchange, *ELR* Electronic laboratory report, *LR* Laboratory report

^a^Source here could be either HIE-ELR or Faxed-LR

## Discussion

Reporting rates along with the completeness and timeliness of notifiable disease case reports, stratified by reporting source, were analyzed for a large local health department in preparation for an intervention to improve provider reporting. Monitoring these ‘vital statistics’ of public health surveillance is important to understanding the quality of data received and used by health departments to measure disease burden and develop interventions to improve population health.

The results illustrate that provider reporting rates, as well as case report completeness and timeliness, have room for improvement. They further identify the opportunity for greater information system integration between clinical and public health organizations, which the federal meaningful use policy initiative may help achieve [32]. Currently the CDC, with support from the Robert Wood Johnson Foundation, is conducting a pilot program to test a ‘digital bridge’ between clinical and public health organizations for notifiable disease reporting in conjunction with the meaningful use program [33].

The observed provider reporting rates were similar to those of previously published studies [4, 6]. We observed that routine infections like chlamydia had higher reporting rates than less common diseases. Some cases of Hepatitis B and Hepatitis C may have been chronic cases that providers’ perceived as low priority, which might explain the low rates for these diseases. The reason for the disjointed reporting of syphilis depicted in Fig. 2 is less clear. An examination of the provider, HIE-ELR and Faxed-LR reports for syphilis cases shows a potential, though unconfirmed, influence based on the affiliation of the ordering provider. Two of the major health system laboratories in the county appear to have relied solely on the Indiana HIE for reporting of syphilis cases; the other health systems appear to have relied solely on fax machines for reporting information on syphilis to the health department.

These observations suggest that provider reporting rates, and variation by disease, are heterogeneous and largely unchanged despite the growth in the adoption and use of EHR systems as well as numerous communications from public health agencies to providers on the importance of reporting notifiable conditions. There is therefore opportunity for our planned intervention to improve reporting by leveraging an HIE infrastructure to pre-populate and electronically deliver provider case reports. Similarly, initiatives like the Digital Bridge for electronic case reporting [18, 33] may impact reporting rates. Evaluation of interventions like the digital bridge and our pre-populated report module will be necessary to determine effectiveness in real-world public health settings.

Like reporting rates, the completeness of data within submitted reports also has room for improvement. Rates varied across data fields ranging from almost always incomplete (e.g., ethnicity in HIE reports) to 100% complete (e.g., patient’s name). We further observed variation based on the source of the submitted reports. Sometimes laboratory-based reports, whether faxed-LR or HIE-ELR, were more complete for some fields (e.g., patient sex, lab test performed), whereas other fields (e.g., ethnicity) were more complete in provider reports. These findings are consistent with our prior work on improving data completeness in ELR [30, 31].

There exists a temptation to conclude that, given the strength of completeness with respect to laboratory reports, public health agencies might abandon provider reports as a source of data for surveillance. However, provider reports often contain information not available from the lab, including documentation of whether or not the patient is receiving treatment. Moreover, we found that some provider report fields such as patient address, patient zip code, race and ethnicity were significantly more complete than equivalent fields in laboratory reports. Therefore, dual-reporting continues to be important for accurate surveillance of notifiable diseases, and interventions that can improve provider-based data completeness are worthwhile to explore.

The timeliness with which electronic reports are received at the health department indicates an advantage of integrated information systems. Electronically submitted laboratory reports were generally available at the health department within 24–48 h of the positive laboratory result; a finding similar to those from prior ELR studies [34–36]. Provider reports, which are usually faxed to public health departments, arrived about a week after the diagnosis. This delay is likely due to the clinical workflow associated with public health reporting. Following a confirmed diagnosis by the laboratory, clinic staff must 1) be notified of the result; 2) communicate the result to the patient; 3) prescribe treatment to the patient; 4) assign the public health report to a member of the clinical team (e.g., nurse, medical assistant); and 5) submit the completed report to the health department. Furthermore, per comments from local health department staff, the delay may also be impacted by the “phone tag” that disease investigators play with clinic staff to remind them to complete the form.

Our intervention will pre-populate a case reporting form and deliver it to a provider in parallel with the laboratory result. The pre-populated form may act as a reminder that the case should be reported to public health. Furthermore, when providers review the pre-populated form, they may notice that sections on treatment and other important data fields require their attention. We hypothesize this will lead to more complete and timely information in provider reports in addition to increasing the reporting rate from providers who receive the intervention.

One limitation of the current analysis is the varying time periods for which data were captured from the health department. Because reports for some diseases were captured over a longer time horizon, it is possible that unmeasured factors such as workflow or technological change during the longer period may have influenced the completeness or timeliness of submitted reports. When we measure the impact of the intervention, we will use similar timeframes to control for this potential source of confounding.

## Conclusions

Receipt of complete, timely information is critical to the work of public health. When disease investigators receive incomplete information, they must call providers’ offices or track down details through time-consuming and complex medical records review processes. This is burdensome, and costly, for both clinical and public health organizations. Moreover, when information is not reported at all, estimates of disease burden or predictions of future disease trends will be inaccurate.

Our intervention will seek to make it easier for the provider to do the right thing. Moving forward, we will analyze the impact of pre-populated forms intervention on reporting rates, data completeness, clinical staff burden and timeliness to further understand how EHR systems and HIE networks can support improved, efficient public health surveillance processes.

## Abbreviations

CDC: Centers for Disease Control and Prevention
EHR: Electronic health record
ELR: Electronic laboratory reporting
HIE: Health information exchange
LR: Laboratory report
